# Acidification and Calcium Addition Effects on High-Pressure and Thermally Induced Pulse Protein Gels

**DOI:** 10.3390/gels11120971

**Published:** 2025-12-02

**Authors:** April Huang, Carmen I. Moraru

**Affiliations:** Department of Food Science, Cornell University, Ithaca, NY 14853, USA

**Keywords:** pulse proteins, high pressure processing (HPP), thermal treatment, gelation, rheology, structure, protein interactions

## Abstract

Modulating the characteristics of pulse protein gels provides opportunities for creating gelled products with unique structures and textures. This work investigates the effects of acidification (pH of 6.3–6.6, 5.5, 4.5), calcium addition (0–30 mg Ca/g protein), and process type (nonthermal vs. thermal) on the structural characteristics of gels made from pea, lentil, and faba bean protein concentrates. Protein concentrate suspensions were processed under conditions that lead to gel formation, either by high-pressure processing (HPP) at 600 MPa, 5 °C for 4 min, or thermal processing at 95 °C for 15 min. The resulting gels were evaluated for rheological properties, texture, water holding capacity, and structure. Both acidification and calcium addition increased protein aggregation due to reduced electrostatic repulsion among protein molecules. Acidification increased the strength of both HPP- and thermally induced gels, while the effect of calcium addition depended on pH and process type. Generally, HPP-induced gels had lower mechanical strength than thermally induced gels, but certain combinations of acidification and calcium addition produced HPP-induced gels stronger than their thermally induced counterparts. These results demonstrate how the structure and mechanical properties of pulse protein gels can be customized through a combination of acidification, calcium addition, and processing. This approach can be used as a foundation for the development of plant protein-based foods of desired structure and texture.

## 1. Introduction

With the rise in demand for plant protein-based food products with high nutritional and functional attributes, proteins from pulses, which are the dry seeds of leguminous crops such as peas, beans, and lentils, are attractive candidates for food applications due to their low ecological footprint and cost [1]. A common category of pulse ingredients is represented by pulse protein concentrates produced by air classification, with a protein concentration of 50–65% and some starch content [1]. Compared to protein isolates, concentrates retain the native functionality of proteins and avoid the use of solvents [2].

Protein gelation, in which proteins form three-dimensional matrices that hold water and provide mechanical strength, is the basis of many semi-solid foods such as yogurt, cheese, and meat analogs. The characteristics of protein gels depend on a multitude of factors, such as the denaturing force, pH, and the presence of salt ions [3]. The appropriate manipulation of the factors that affect the gelation of pulse protein concentrates can open opportunities for the development of novel plant-based food products with gel structure.

To induce gelation, a denaturing force that results in protein unfolding is applied, which leads to the exposure of initially buried hydrophobic groups, new molecular interactions, and subsequent aggregation [4]. Gelation can be achieved using both physical and chemical drivers. A common physical driver is thermal processing, which is used in many gelation applications. In recent years, high-pressure processing (HPP) has been identified as a nonthermal treatment able to induce protein gelation. While HPP is primarily used for the inactivation of vegetative microorganisms, pressure levels typically used in HPP (400–600 MPa) can also modify protein structures and create gels. Compared to thermal processing, HPP has the advantages of a short treatment time, treatment uniformity, and the preservation of heat-sensitive compounds, and it avoids the development of off-flavors common in heated pulse products [5,6]. HPP-induced gelation produces distinct gel characteristics compared to thermal processing. HPP-induced gels are typically smoother, more homogenous, and less brittle, but have lower gel strength and water holding capacity compared to thermally induced gels [7,8,9]. The level of pulse protein denaturation was found to be similar after both processes, but mechanistic differences in HPP- vs. thermally-induced gelation impact the protein interactions and resulting gel properties [3,9]. During thermal processing, weak interactions are broken due to increasing molecular vibration; during HPP, the shift in equilibrium towards reduced volume states results in the disruption of protein structures through the (i) compression of protein cavities to eliminate packing defects, (ii) dissociation of ion pairs to decrease solvent volume, and (iii) destabilization of hydrophobic interactions [10,11,12,13]. The shortening of hydrogen bonds, the strengthening of van der Waals forces, and to some extent the formation of new disulfide bonds, can serve to stabilize the denatured proteins [10,14].

Since HPP-induced gels are mostly stabilized by noncovalent interactions and thus have low mechanical strength, it is important to seek solutions to increase their strength and stability. Therefore, the aim of this research was to use acidification and calcium addition to achieve this. Both acidification and calcium fortification are used in food production for safety and quality reasons. Acidulants like glucono-δ-lactone (GDL) and salts such as calcium chloride (CaCl_2_) are commonly used as coagulants in the production of milk and soy protein-based gelled foods, since they diminish the repulsive forces among protein molecules and facilitate protein–protein interactions [15]. As the pH approaches the isoelectric point (pI) of proteins, which for pulse proteins ranges between pH 4–5, protein molecules near a net charge of zero, facilitating their interaction [16]. It is also important to note that the acidification to pH ≤ 4.6 is often used for HPP products as a food safety hurdle, to control foodborne pathogens [17]. Cations like Ca^2+^ make a cloud around the protein molecules, decreasing the stabilizing repulsion between them [18]. As a divalent cation, Ca^2+^ can also cross-link adjacent anionic groups in protein molecules, stabilizing protein networks by reinforcing existing and/or establishing new interactions [19].

This study investigates the effects of calcium addition, acidification, and processing method (thermal vs. HPP) on the gelation of pulse protein concentrates. Processing was conducted under conditions that were previously identified to result in pulse protein denaturation and gel structure formation and are practical for industrial applications [9,20,21,22,23]. The generated knowledge is expected to offer pathways that lead to a range of textures in pulse protein concentrates through a combination of processing and formulation. This approach can then be used as a foundation for developing plant protein-based foods of desired structure and texture, which can help the food industry meet consumer demand for high-quality, plant protein food products.

## 2. Results and Discussion

### 2.1. Properties and Stability of Pulse Protein Concentrate Suspensions Prior to Processing

**Figure 1:**
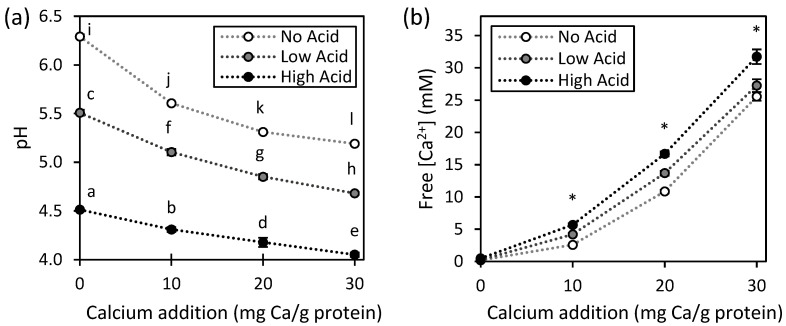
(**a**) pH and (**b**) free Ca^2+^ concentration of unprocessed PPC suspensions at different acidification and calcium addition levels. Values represent averages of independent biological triplicates, which are each an average of technical triplicates. Error bars represent ±1 standard error. Different letters represent significant differences in pH and * indicates significant differences between the free [Ca^2+^] at No Acid, Low Acid, and High Acid, at a given calcium addition level.

The initial pH of the three types of protein suspensions ranged between 6.3 and 6.6. As seen in Figure 1a, which shows the data for pea protein concentrate (PPC), the chosen levels of acidification lowered the pH to 5.5 (Low Acid) and 4.5 (High Acid), respectively, approaching the isoelectric point of pulse proteins (pH 4–5). The addition of calcium up to 30 mg Ca/g protein progressively decreased the pH of PPC suspensions for all acidification levels (No Acid, Low Acid, High Acid). The same was found in both lentil protein concentrate (LPC) and faba bean protein concentrate (FPC) suspensions (Supplemental Figure S1). Since the addition of CaCl_2_ to water without added protein concentrate, at the same concentration levels as in the protein suspensions, resulted in a slight increase in alkalinity (by approximately 0.5–1 pH units), the observed pH decrease in suspensions is likely due to interactions between calcium ions with protein functional groups. According to previous reports, Ca^2+^ and H^+^ compete for the same anionic binding sites in the phosphate esters of phytates from legumes, as well as the carboxyl groups of aspartic and glutamic acid residues and imidazole groups of histidine residues of the pulse protein molecules, which results in H^+^ being released in solution [24,25,26]. Therefore, any effect of CaCl_2_ addition on protein gelation will likely be due both to calcium and the drop in pH [27].

Figure 1b shows the concentration of free Ca^2+^ in PPC suspensions. At the same level of calcium addition, No Acid samples had the lowest free [Ca^2+^]. This likely happened because Ca^2+^ ions have a high affinity to negatively charged protein binding sites at the unmodified pH of the solution (pH 6.3) [26]. With increasing acidification, some negatively charged binding sites in the protein chains were neutralized, the affinity of Ca^2+^ to these binding sites lowered, and, consequently, free [Ca^2+^] increased. This effect of the competition between Ca^2+^ and H^+^ on free [Ca^2+^] was also observed for the acidification of milk proteins [28].

**Figure 2:**
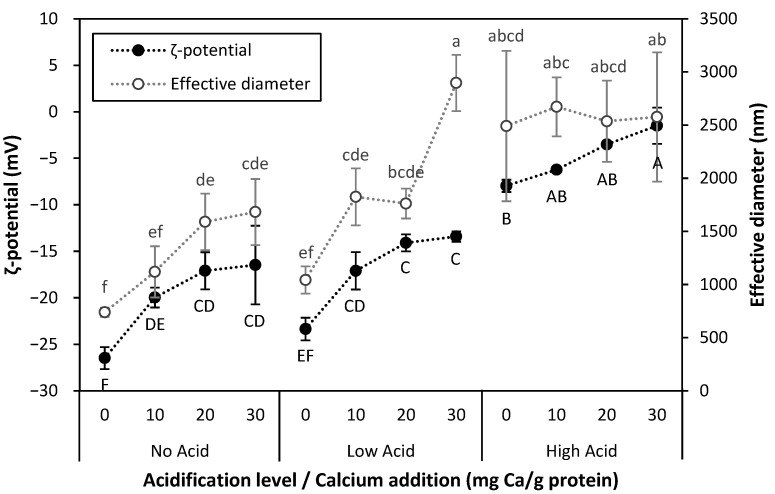
ζ-potential (●) and effective diameter (○) of unprocessed PPC suspensions at different acidification and calcium addition levels. Values represent averages of independent biological triplicates, which are each an average of technical triplicates. Error bars represent ±1 standard error. Different uppercase letters represent significant differences in ζ-potential while different lowercase letters represent significant differences in particle size.

The stability of proteins prior to processing was assessed by determining their zeta potential (ζ-potential) and particle size distribution. For the unmodified PPC suspension (No Acid-0 mg Ca/g protein), as seen in Figure 2, the highest magnitude of the ζ-potential (−26.48 mV) and thus the highest stability, was observed at a pH farthest above the isoelectric point of pea proteins, which is in the pH range 4.0–5.0 [16]. Under these conditions, the strong electrostatic repulsion between protein particles prevented their aggregation, as supported by this sample having the lowest effective diameter (702 nm) [27]. The magnitude of ζ-potential progressively dropped with acidification and calcium addition, as H^+^ and Ca^2+^ ions screened negatively charged protein groups. The decrease in electrostatic repulsive interactions between protein molecules enhanced the relative strength of attractive interactions, such as short-range van der Waals forces, and promoted the formation of increasingly larger protein aggregates [29]; this hypothesis is supported by the increase in particle size. The increase in particle size is illustrated by both the increase in effective diameter (Figure 2) and the shift of the smallest particle sub-population observed around 100 nm towards larger sizes (Supplemental Figure S2). The exception to this trend was represented by the High Acid samples, for which calcium addition reduced the magnitude of ζ-potential but did not increase particle size. The reason may be that, at High Acid, an excess of H^+^ outcompeted Ca^2+^ for binding at negatively charged protein binding sites and thus reduced the ability of Ca^2+^ to form bridges between protein molecules.

Similar effects of acidification and calcium addition were found for LPC and FPC, for which ζ-potential, particle size, and free [Ca^2+^] data is included in Supplemental Tables S1–S3.

### 2.2. Effect of Process Type, Acidification, and Calcium Addition on the Rheological Properties and Microstructure of Pulse Protein Concentrate Gels

**Figure 3:**
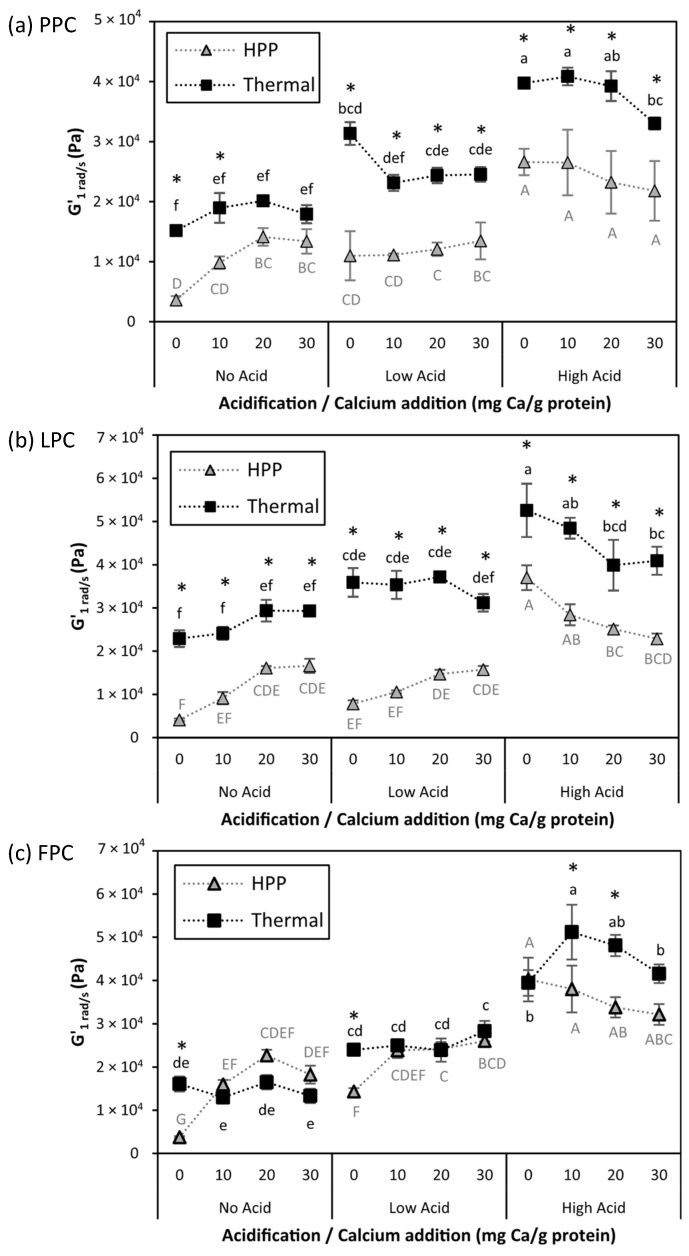
Storage modulus at a frequency of 1 rad/s (G′_1 rad/s_) for (**a**) PPC, (**b**) LPC, and (**c**) FPC gels at different acidification and calcium addition levels, that were thermally processed (95 °C, 15 min; ◼) or HPP-treated (600 MPa, 5 °C, 4 min; 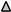). Values represent averages of independent biological triplicates, which are each an average of technical triplicates. Error bars represent ±1 standard error. Different uppercase and lowercase letters indicate significant differences within HPP- and thermally processed samples, respectively. Within the same acidification and calcium addition level, * indicates significant difference between process types. PPC-High Acid-0 mg Ca/g protein has biological duplicates due to a sample loss.

The rheological properties of the samples were used to assess the structural changes induced by each treatment. Elastic modulus (G′), which is a measure of the strength of the material and denotes solid-like behavior, viscous modulus (G″), which represents the liquid-like behavior, and loss tangent (tan δ) were obtained from small amplitude, oscillatory frequency sweeps. The linear viscoelastic range (LVR) was first determined using strain sweeps (see Supplemental Figure S3), after which frequency sweeps were conducted at strain values in the LVR. Supplemental Figure S4 shows the G′ vs. frequency for all samples. To compare gel stiffness across different acidification and calcium addition levels, G′ values at a frequency of 1 rad/s (G′_1 rad/s_) of all processed samples were extracted and are shown in Figure 3.

**Figure 4:**
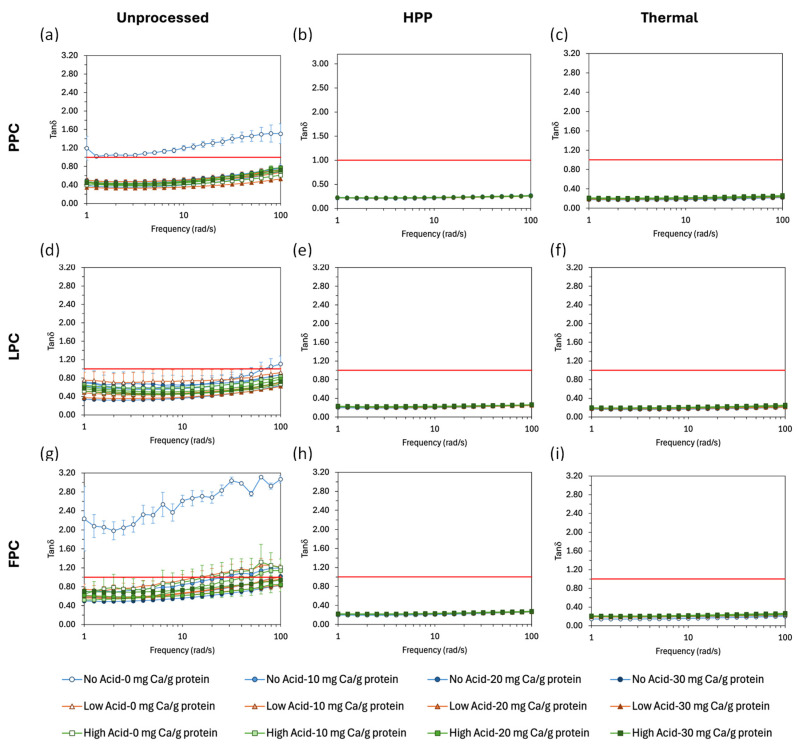
Tan δ vs. frequency for unprocessed, HPP-, and thermally processed PPC (**a**–**c**), LPC (**d**–**f**), and FPC (**g**–**i**) samples at different acidification and calcium addition levels. Values represent averages of independent biological triplicates, which are each an average of technical triplicates. Error bars represent ±1 standard error. Horizontal red lines indicate tan δ = 1 (G′ = G″).

Prior to processing, samples displayed liquid or weak gel behavior, with G′ values of 10^1^–10^2^ Pa. Of unprocessed suspensions, No Acid-0 Ca/g protein samples had the lowest G′ values across all frequencies, and generally tan δ > 1 (Figure 4), which indicates a predominantly liquid-like behavior with largely unassociated particles; this was expected, given the large magnitude of the ζ-potential, conducive of strong electrostatic repulsion. With acidification and/or calcium addition, due to the increase in attractive interactions and protein aggregation, G′ increased and most samples had tan δ < 1, indicating the formation of a weak gel. HPP and thermal processing both produced self-standing gels, with a higher magnitude of G′ (10^3^–10^4^ Pa) and tan δ < 1 over the entire frequency range.

**Figure 5:**
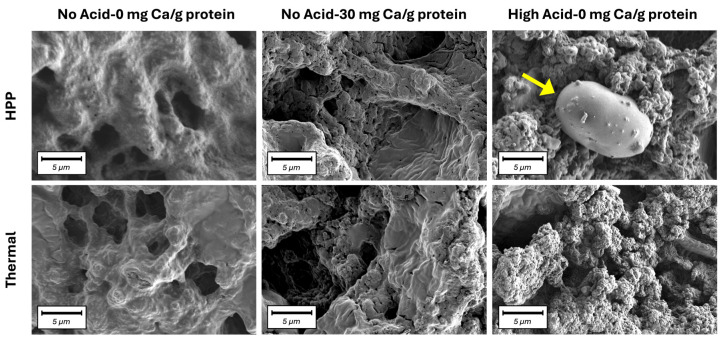
Scanning electron microscopy (SEM) images of HPP- and thermally processed PPC gels at No Acid-0 mg Ca/g protein, No Acid-30 mg Ca/g protein, and High Acid-0 mg Ca/g protein. Scale bars represent 5 µm. The yellow arrow indicates a starch granule.

Across all protein types, thermally induced gels had similar or higher G′_1 rad/s_ values compared to their HPP-induced counterparts. Tan δ values were also generally lower in thermally induced gels (0.14–0.27) than HPP-induced gels (0.19–0.28), indicating that the former had stronger solid-like behavior. This is in agreement with prior work from our lab [9], which showed that thermal processing created stronger PPC, LPC, and FPC gel networks than HPP, despite a similar degree of protein denaturation. Others also showed that, for both soybean protein isolates [30,31,32] and cowpea protein [33], thermally induced gels were stronger than HPP-induced gels. Thermal processing creates stronger gel networks than HPP, primarily due to strong intermolecular linkages that can form due to a greater extent of exposure of reactive groups [34,35]. In the case of the protein concentrates used in this work, a secondary contributor could have been the gelatinization of the significant starch fraction under thermal processing, which reinforced the overall network [34]. In HPP gels, starch granules remain intact and behave as an inert filler [21]. Intact starch granules, with a smooth kidney-shaped appearance, were observed in SEM images of HPP PPC gels, as shown in the HPP High Acid-0 mg Ca/g protein sample in Figure 5 (higher magnification) and Supplemental Figure S5 (lower magnification).

Overall, gel strength increased with acidification. At 0 mg Ca/g protein, the G′_1 rad/s_ values of both thermally processed and HPP gels were the lowest at No Acid and highest at High Acid. With reduced electrostatic repulsion caused by the addition of acid, attractive forces between protein molecules dominated, facilitating aggregation and the formation of a stronger, more interconnected, gel network. The SEM micrographs (Figure 5, Supplemental Figure S5) and the photos of gels in Supplemental Figure S6 show that the High Acid-0 mg Ca/g protein sample was coarser and more granular than the No Acid-0 mg Ca/g protein sample.

The effect of calcium addition was dependent on both process type and acidification level. Under No Acid conditions, thermally induced gels showed no significant changes in G′_1 rad/s_ with calcium addition. This differs from previous reports that indicated that calcium addition strengthened thermally induced gels made from pea protein isolate [27], soy protein isolate [36], and pea, lentil, and faba bean protein concentrates [37]. A key distinction between this work and these previous studies is that the pulse protein concentrates used here were produced via air classification, which largely preserves the native protein structure [32], whereas protein materials in previous studies were produced under denaturing conditions (alkaline extraction and isoelectric precipitation). The pre-denaturation of proteins may expose additional binding sites for calcium, facilitating more extensive calcium bridging during heating compared to the present study, which used undenatured proteins. Future work will be needed to confirm this hypothesis.

By contrast, the No Acid HPP gels for all protein types had significantly higher strength (G′_1 rad/s_) at 20 and 30 mg Ca/g protein than at 0 mg Ca/g protein, suggesting that calcium addition enhanced protein aggregation, likely due to the formation of calcium-mediated bridges between protein chains. Previous studies reported that high-pressure conditions promote the transient dissociation of calcium ions from protein binding sites [12], allowing them to rebind in configurations that favor cross-linking between unfolded protein chains, ultimately strengthening the gel network [38,39]. However, with increasing acidification, the positive effect of calcium addition on HPP-induced gel stiffness diminished. At Low Acid, calcium addition only increased G′_1 rad/s_ in FPC HPP gels and had no significant impact on the other samples. At High Acid, increasing calcium addition had either no effect or a detrimental effect on the strength of both HPP- and thermally induced gels. G′_1 rad/s_ values were lowest in thermally processed PPC samples at 30 mg Ca/g protein, and in both thermally processed and HPP LPC gels at 20 and 30 mg Ca/g protein. Manassero et al. [38], who studied the effect of minerals during the HPP of soy protein isolate (likely in a pre-denatured state), hypothesized that, at high binding affinity, Ca^2+^ may be buried in the binding site of a single protein, preventing it from making bridges between proteins, while at a low affinity Ca^2+^ may be more available to participate in intermolecular bridges. Our observations do not align with this theory. For the (undenatured) proteins used in the current study, it appears that the higher affinity of Ca^2+^ to anionic sites on the protein chains without acidification favored the formation of bridges, whereas the decreased calcium binding affinity at higher acidification levels reduced its ability to participate in bridging. This aligns with previous observations in dairy systems, where the solubilization of calcium with acidification reduces calcium bridging between caseins [28].

The complex influence of acidification and calcium addition and their interactions on gel stiffness are thought to reflect the balance of two opposing contributions. On the one hand, the reduction in repulsion and increase in protein–protein interactions increased gel stiffness; on the other hand, localized protein aggregation can result in fewer connection points in the network and a subsequent weakening of the gel matrix [40,41,42]. This possibility is supported by the larger voids observed in the microstructure of samples that were acidified and/or had calcium compared to the No Acid-0 mg Ca/g protein sample (Supplemental Figure S5).

### 2.3. Effect of Process Type, Acidification, and Calcium Addition on the Textural Properties and Macrostructure of Pulse Protein Concentrate Gels

#### 2.3.1. Gel Cohesiveness and Hardness

**Figure 6:**
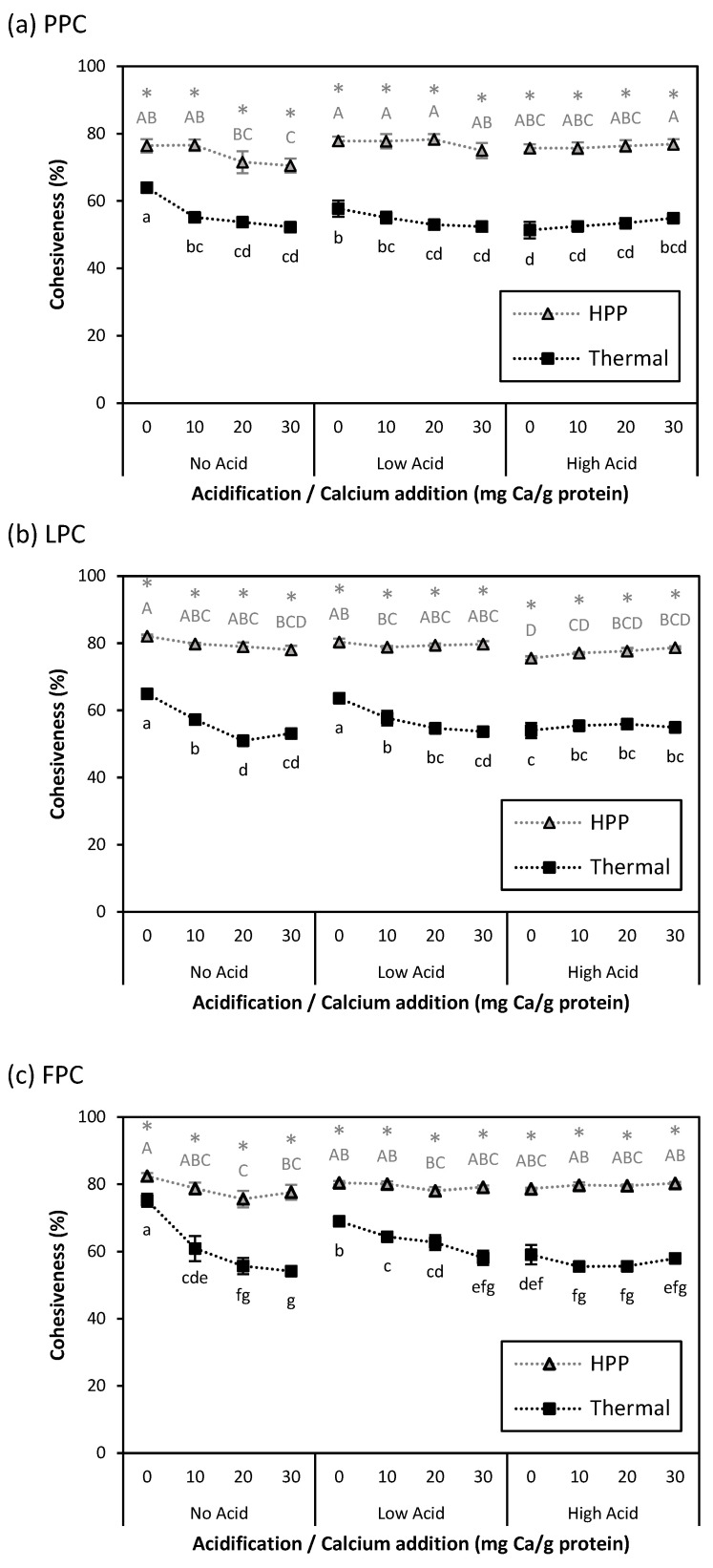
Cohesiveness for (**a**) PPC, (**b**) LPC, and (**c**) FPC gels at different acidification and calcium addition levels for thermally processed (95 °C, 15 min; ◼) or HPP treated (600 MPa, 5 °C, 4 min; 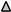) samples. Values represent averages of independent biological triplicates, each being an average of technical triplicates. Error bars represent ±1 standard error. Different uppercase and lowercase letters indicate significant differences within HPP- and thermally processed samples, respectively. Within the same acidification and calcium addition level, * indicates significant difference between process types. PPC-High Acid-0 mg Ca/g protein, PPC-High Acid-10 mg Ca/g protein, PPC-High Acid-20 mg Ca/g protein had only biological duplicates, due to a sample loss.

**Figure 7:**
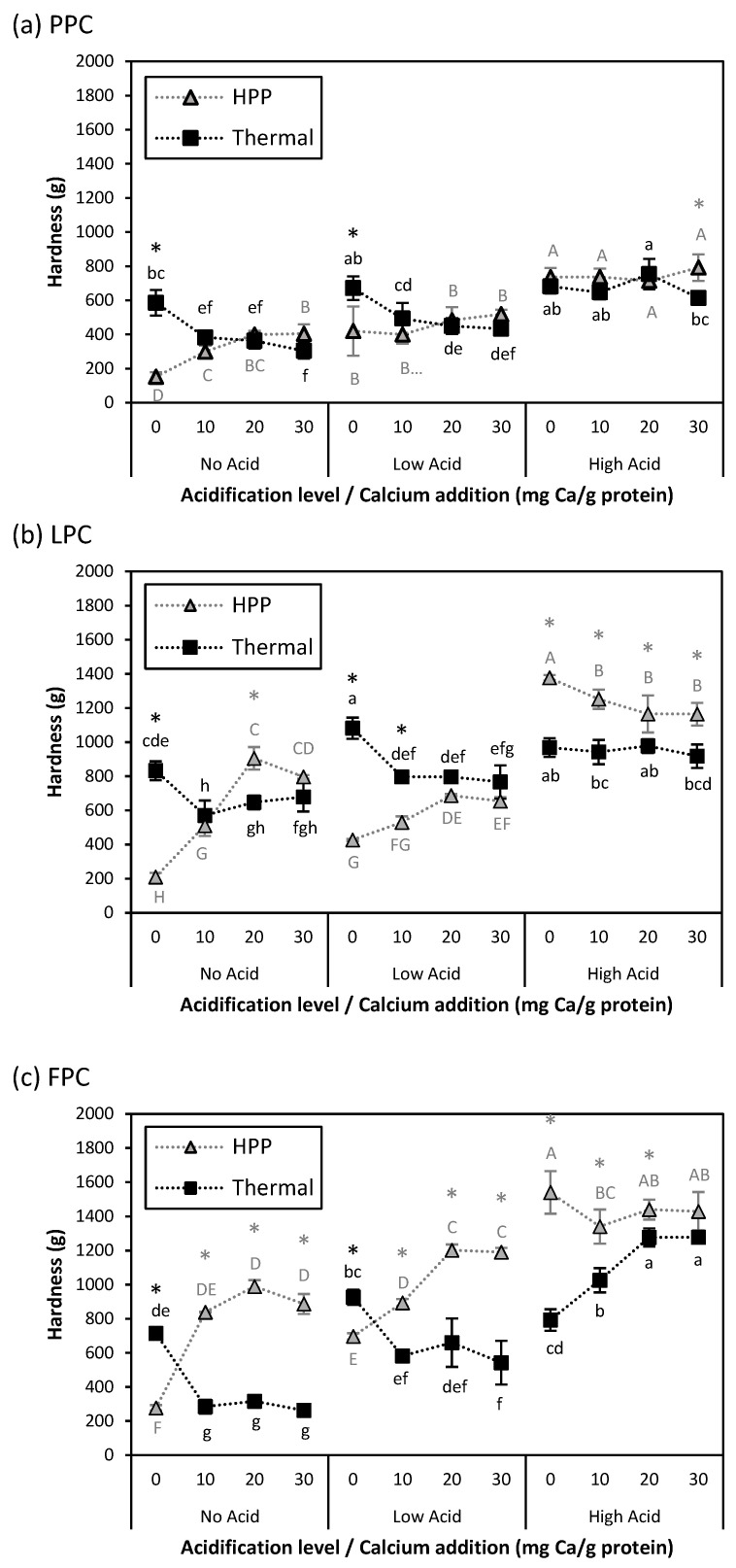
Hardness values for (**a**) PPC, (**b**) LPC, and (**c**) FPC gels at different acidification and calcium addition levels for samples that were thermally processed (95 °C, 15 min; ◼) or treated by HPP (600 MPa, 5 °C, 4 min; 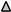). Values represent averages of independent biological triplicates, which are each an average of technical triplicates. Error bars represent ± 1 standard error. Different uppercase and lowercase letters indicate significant differences within HPP- and thermally processed samples, respectively. Within the same acidification and calcium addition level, * indicates significant difference between process types. PPC-High Acid-0 mg Ca/g protein, PPC-High Acid-10 mg Ca/g protein, PPC-High Acid-20 mg Ca/g protein have biological duplicates due to a sample loss.

While the rheological data discussed above provides useful information about sample microstructure and the strength of internal bonds, the way samples will behave during processing or mastication is more accurately indicated by texture analysis. The texture parameters cohesiveness and hardness of HPP- and thermally induced gels are presented in Figure 6 and Figure 7. Before discussing these properties, a note should be made about the presence of air bubbles inside the processed samples, as they can have a significant impact on their texture. Although all protein suspensions were degassed prior to processing, the weak gel structure formed after acidification and calcium addition facilitated the retention of air bubbles. Images of processed samples indicate coarseness and the presence of visible air bubbles in the thermally induced gels, compared to the smoother and more uniform HPP-treated samples. While heating promoted the coalescence of air bubbles by increasing kinetic energy, pressurization compressed entrapped air bubbles, making them smaller. This is consistent with a previous report that has shown that HPP treatment resulted in a more uniform distribution of smaller bubbles in cake batter compared to an untreated control [43]. Samples with higher acid addition also had a coarser appearance compared to those with no or low acid addition (Supplemental Figure S6).

Across all acidification and calcium addition levels, HPP-induced gels were significantly more cohesive than thermally induced gels. HPP-induced gels had greater resilience to compression than thermally induced gels because the volume reduction in intra- and inter-protein cavities and uniformity of protein denaturation with the isotropic transfer of pressure during HPP produced a denser, more homogenous gel matrix. In thermally induced gels, acidification and calcium addition further decreased cohesiveness, likely due to the disruption of the gel matrix by air pockets.

The impact of acidification and calcium addition on gel hardness also depended on process type. Acidification alone increased hardness in all HPP-induced gels, and in thermally induced LPC and FPC gels between No Acid and Low Acid. Any calcium addition at No Acid or Low Acid decreased the hardness of thermally induced gels but increased the hardness of HPP-induced gels (except for Low Acid PPC gels). Weaker gels at similar levels of calcium addition were also observed in heat-induced quinoa protein and whey protein gels [42,44]. Tomczyńska-Mleko et al. (2015) reported that the increased protein aggregation caused by high concentrations of cations (such as Ca^2+^) in a whey protein gel system resulted in higher surface roughness and aerated gels [45]. As discussed in the previous section, calcium addition may increase HPP-induced gel hardness due to the formation of calcium bridges between proteins. At High Acid, the effect of calcium addition on hardness was less clear: thermally induced FPC gels and HPP-induced LPC gels with calcium addition had higher and lower hardness, respectively. As with G′_1 rad/s_, this could be due to reduced calcium bridging upon acidification. Calcium addition had no impact on other treatment combinations. The increased coarseness in the macroscopic appearance of thermally induced gels appeared to correspond with lower hardness. Comparing the macroscopic appearance of FPC gels (Supplemental Figure S6), thermally induced No Acid-30 mg Ca/g protein and High Acid-0 mg Ca/g protein gels had a coarser appearance with larger voids than No Acid-0 mg Ca/g protein and High Acid-30 mg Ca/g protein gels, or their HPP-induced counterparts, and also had lower hardness.

Although No Acid-0 mg Ca/g protein HPP-induced gels had lower hardness than their thermally induced counterparts, acidification and calcium addition increased their hardness to the same level as, or even above, that of thermally induced gels, due to their effects on protein interactions.

#### 2.3.2. Gel Water Holding Capacity and Syneresis

**Figure 8:**
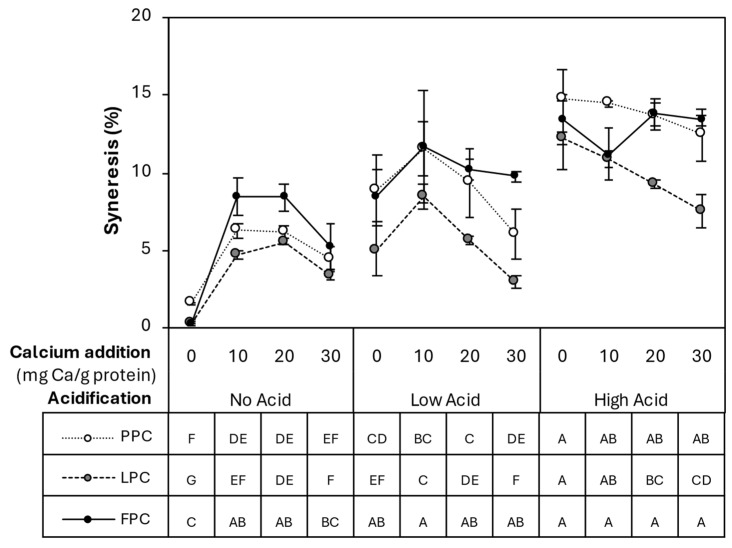
Syneresis (%) of HPP PPC, LPC, and FPC samples, measured 1 h after processing. Values represent averages of independent biological replicates and error bars represent ±1 standard error. LPC samples were prepared in biological triplicates, whereas PPC and FPC samples were prepared in biological duplicates due to limited materials. Each independent replicate was measured in technical duplicates. Different letters indicate significant differences within each protein type. Note: Thermally processed samples showed no syneresis and thus are not included in this graph.

Based on visual observations, thermally induced gels had no obvious syneresis following processing, whereas all HPP-induced samples displayed some level of water separation. This exclusion of water was likely due to the contraction of the gel matrix with the collapse of intermolecular cavities during pressurization. The syneresis data for HPP samples is shown in Figure 8. Syneresis was lowest in No Acid-0 mg Ca/g protein samples (<2% of the original sample weight) but ranged from 3 to 15% in other samples. Both acidification and intermediate levels of calcium (10–20 mg Ca/g protein) increased aggregation. However, in both PPC and FPC gels at No Acid, syneresis at the highest calcium level (30 mg Ca/g protein) was lowered to values not significantly different from the No Acid-0 mg Ca/g protein samples. Although syneresis in HPP-induced gels may pose a challenge to product quality and consumer acceptance, this suggests that identifying formulation-based solutions, such as sufficient calcium, may be able to mitigate this issue.

Water holding capacity was used as a proxy for the propensity of samples to develop syneresis over shelf-life. Gels with lower water holding capacity can experience shrinkage and changes in texture with prolonged storage. As shown by the water holding capacity values in Table 1, thermally induced gels had the highest water holding capacity (~100%), followed by HPP gels (87–97%) and unprocessed suspensions (35–80%). Both thermally and HPP-induced gels had significantly higher water holding capacity compared to unprocessed samples due to the entrapment of water by the protein network. HPP samples had significantly lower water holding capacity than thermally processed samples for FPC, but the difference between process types was mostly insignificant for PPC and LPC. Thermally induced gels likely hold more water than HPP-induced gels due to the formation of hydrogen bonds between starch and water during starch gelatinization [46].

Although both acidification and calcium addition increased the water holding capacity of samples in unprocessed suspensions due to the formation of weak gel structures, they had no clear influence on the water holding capacity of either HPP- or thermally induced gels. These results indicate that, although HPP-induced gels showed an initial expulsion of water immediately after processing, the water holding stability of these gels was stable over shelf-life, with values not far below those of thermally induced gels.

A summary of the main trends of the effects of acidification and calcium addition on the properties of HPP- and thermally induced pulse protein concentrate gels is presented in Table 2. For HPP gels, acidification, calcium addition, and their combination consistently enhanced gel strength and hardness. In contrast, thermally processed samples showed increased gel strength but did not change or just slightly increased hardness with acidification, while calcium alone had little effect on gel strength and even reduced hardness. Except for the acidified PPC and FPC HPP gels, which showed no significant difference in cohesiveness compared to their respective controls, acidification and calcium addition generally decreased the cohesiveness of other gels. Acidification also increased post-processing syneresis in all HPP gels, while calcium addition increased syneresis in LPC gels.

## 3. Conclusions

With growing consumer demand for clean label, nutritious, plant-based food products, understanding how processing and formulation impact texture is critical. While thermal treatment is typically used for processing such products, high-pressure processing offers an alternative for applications where the use of heat is undesirable. This work demonstrates that acidification and calcium addition, in combination with either HPP or thermal processing, can be used to create pulse protein gels ranging from soft to strong gels. Thermally induced gels were generally stronger, while HPP-induced gels exhibited greater cohesion. Acidification near the isoelectric point of pulse proteins increased the strength and hardness of both thermally and HPP-induced gels, making it a promising strategy for the manufacture of acidified products such as yogurt- or cheese/tofu-like products. HPP-gels were strengthened by calcium addition, but showed syneresis immediately after processing, which needs to be addressed in future product development applications. Overall, the results of this work demonstrate that the structure and mechanical properties of pulse protein gels can be fine-tuned by optimizing protein aggregation through processing (by pressure or heat) and formulation (by adding acid or calcium). These findings can inform the development of novel plant protein foods from sustainable pulse protein ingredients, using treatments that combine structural and textural benefits with food safety.

## 4. Materials and Methods

### 4.1. Materials

Pulse protein concentrate powders manufactured by air classification [VITESSENCE Pulse 1550 (yellow pea), 2550 (lentil), 3600 (faba bean) protein concentrate powders] were provided by Ingredion (Bridgewater, NJ, USA). These are referred to as PPC (pea protein concentrate), LPC (lentil protein concentrate), and FPC (faba bean protein concentrate). The composition of the protein powders, determined by Dairy One Laboratories (Ithaca, NY, USA), is provided in Table 3.

Glucono *δ*-lactone (GDL, Thermo Scientific Chemicals, Ward Hill, MA, USA) and anhydrous calcium chloride (CaCl_2_, Occidental Chemical Corp., Dallas, TX, USA), and water produced using a Milli-Q^®^ water purification system (Millipore Sigma, Burlington, MA, USA), were used for sample preparation.

### 4.2. Preparation of Protein Concentrate Suspensions

Stock protein concentrate suspensions (18–19 g protein/100 g) were prepared by mixing PPC, LPC, and FPC powders into water (Thermomix TM6, Vorwerk, Wuppertal, Germany) for 10 min. Suspensions were then high shear mixed (UltraTurrax Model T25, IKA Works Inc., Wilmington, NC, USA) and stored at 4 °C for hydration overnight (16–20 h). Following hydration, glucono-δ-lactone (GDL) was incorporated (0, 45, 165 mg GDL/g for pea protein and 0, 50, 170 mg GDL/g lentil and faba bean protein) under continuous stirring over 2 min. A final protein concentration of 15 g/100 g was chosen for its relevance to solid gel food products such as tofu and cheese and because this protein concentration demonstrated strong gel formation in work by Hall and Moraru [9]. The composition of the protein suspensions is presented in Table 4.

The levels of GDL addition were established in preliminary work to achieve a starting pH of 4.5 ± 0.1 and 5.5 ± 0.1. These pH conditions were chosen due to their impact on the net charge of proteins, since the isoelectric point (pI) of pulse proteins is between pH 4 and 5 [1]. As calcium (as CaCl_2_) addition also impacted pH, the acidification levels were referred to as No Acid, Low Acid, and High Acid rather than by the pH values. GDL was chosen as the acidulant to avoid the instantaneous protein precipitation that occurs with strong acid addition. Its slow hydrolysis to gluconic acid ensures a gradual and progressive pH decline.

Calcium was then added through CaCl_2_ addition at 0, 10, 20, or 30 mg Ca/g protein. CaCl_2_ was chosen as the Ca^2+^ source due to its prevalent use in the food industry and higher solubility compared to calcium sulfate, another common coagulant. As pH is influenced by both GDL and CaCl_2_ addition, it was treated as a response variable, while acidification and calcium addition were selected as independent variables to reflect the practical formulation choices in food systems and capture the interaction of the two factors on gel properties. Additional water was used, as appropriate, to equalize final sample masses and achieve the target protein concentration of 15 g/100 g protein, GDL, and calcium concentrations across all samples. All the subsequent processing of samples was carried out between 4–5 h of acidification, as preliminary work demonstrated that the pH of suspensions after GDL addition reached a steady state at ~4 h.

To prevent or at least minimize the presence of air voids in gels, all protein suspensions were degassed through centrifugation at 1000× *g* for 1 min (Eppendorf Centrifuge 5810, Enfield, CT, USA), skimmed of surface foam, and gently stirred to mix prior to packaging. Unprocessed controls from each batch were stored at 4 °C and analyzed within 2 days of preparation.

### 4.3. High-Pressure Processing

Samples were filled and sealed in cellulose dialysis tubes with a diameter of 27 mm when full (Sigma-Aldrich, St. Louis, MO, USA), than further packaged and heat-sealed in a double layer of poly nylon vacuum bags (Uline, Pleasant Prairie, WI, USA), to withstand the pressurization conditions used in the study. The dialysis tubes were chosen due to their water impermeability and pressure resistance under the conditions used, flexibility—which allows pressure transmission, and cylindrical geometry—convenient for subsequent sample testing. HPP treatment was conducted at 600 MPa for 4 min at 5 °C in a 55 L HPP unit (Hiperbaric, Burgos, Spain) at the Cornell High-Pressure Processing Validation Center (Geneva, NY, USA). These conditions were previously found to result in sufficient protein denaturation, while also being practical for industrial applications [9,20]. Following processing, samples were stored at 4 °C until analysis within 3 days of treatment.

### 4.4. Thermal Processing

Samples were treated within temperature resistant, cylindrical silicone molds (32 mm diameter) at 95 °C for 15 min by immersion in a water bath. These conditions were previously found to be appropriate for pulse protein denaturation and gel structure formation [21,22,23]. Low sample volumes of 20 mL were filled in each mold for efficient heat transfer. Molds were lightly coated in vegetable oil and lined with parchment paper to facilitate sample removal. Following processing, samples were cooled at room temperature for 1 h and then transferred to cold storage at 4 °C until analysis within 3 days of treatment.

### 4.5. Particle Size and Zeta Potential Measurements

The particle size of unprocessed samples was evaluated using a 90Plus Nanoparticle particle size analyzer (Brookhaven Instruments Corp., Holtsville, NY, USA). Measurements were conducted with a 35 mW solid state laser at a fixed 90° angle and a wavelength of 658 nm at 22 °C. To ensure a proper signal intensity and prevent multiple scattering or viscosity effects, samples were serially diluted in filtered (0.2 µm) water to a final concentration of 0.25% (*v*/*v*) to ensure a signal intensity of ~400 kcounts/s. This dilution also reduces the concentration of all species, including calcium ions and protons. The measured sizes of acid- and calcium-induced aggregated systems correspond to the sizes of aggregated particles that do not de-aggregate upon dilution. The refractive indices of water and protein particles were set to 1.33 and 1.45, respectively. The instrument’s built-in software was used to calculate an intensity-weighted effective diameter for each sample. The zeta potential was measured using the ZetaPlus instrument (Brookhaven Instruments Corp., Holtsville, NY, USA).

### 4.6. Free Ca^2+^ Measurements

Free Ca^2+^ concentration was measured using a calcium ion-selective electrode (Orion Calcium Ion Selective Electrode, Thermo Orion, Inc., Chelmsford, MA, USA) to study the extent of binding of Ca^2+^ ions to protein at different acidification levels. Prior to measurements, an ionic strength adjuster was added to all sample solutions to create a uniform background ionic strength and provide more reproducible measurements. Calibration of the electrode with the following Ca^2+^ standards was performed immediately prior to measurements: 0.001, 0.01, 0.1, 0.25 M. All measurements were conducted at 4 °C.

### 4.7. pH Measurements

Measurements of pH in unprocessed samples were conducted for each protein type at 22 °C (Orion Star A214 pH/ISE Meter, Thermo Fisher Scientific, Singapore).

### 4.8. Rheological Analysis

Small amplitude oscillatory shear rheological analysis of unprocessed, HPP-, and thermally processed samples was conducted with an ARES strain-controlled rheometer (TA Instruments, New Castle, DE, USA). For unprocessed samples which were free flowing, a 50 mm diameter Teflon parallel plate configuration with a gap of 1 mm was used. Two mL aliquots of sample were loaded onto the lower plate, with care taken to avoid bubble formation and excess shearing during the loading procedure. An overfill of sample was preferred to avoid air remaining in the gap and the excess sample was trimmed with a metal spatula to prevent disturbing edge effects. For HPP- and thermally processed samples which formed self-standing gels, a 25 mm diameter Teflon parallel plate with an interplaten gap of 2 mm was used. To avoid slip during testing, 120 grit sandpaper (3M, St. Paul, MN, USA) was affixed with double sided tape onto the lower plate. All measurements were performed at a temperature of 4 °C, maintained using the instrument’s Peltier temperature control system. An isothermal chamber enclosing the parallel plates minimized sample dehydration during measurements. Prior to each measurement, samples were subjected to a 60 s relaxation step. Dynamic strain sweeps between 0.01 to 3% strain were first conducted on representative samples, at a frequency of 1 rad/s, to determine the linear viscoelastic range (LVR) and critical strain value. Frequency sweeps from 1 to 100 rad/s were then performed in triplicate for each sample preparation at a strain value within the LVR. The storage modulus (G′), loss modulus (G″), the loss tangent (tan δ = G″/G′) of the samples were monitored. G′ at 1 rad/s (G′_1 rad/s_) were used for comparison between samples.

### 4.9. Texture Profile Analysis

Texture profile analysis of HPP- and thermally processed gels was performed using a TA.XTPlus Texture Analyzer (Stable Micro Systems Ltd., Surrey, UK). Center cores (diameter of 23 mm, height of 8 mm) of gel samples were compressed twice to 25% of their original height with an acrylic probe [diameter of 1.5 in (38 mm)] at a constant crosshead speed of 1 mm/s with a 5 s rest period between compressions. Samples were stored at 4 °C prior to each measurement, which was performed at 20 °C. Hardness (maximum force during the first deformation cycle) and cohesiveness (ratio of positive force area during the second compression to that of the first compression) were computed by the instrument’s software (Stable Micro Systems Ltd., Surrey, UK) and used for comparisons among samples.

### 4.10. Water Holding Capacity

Water holding capacity was measured based on the methodology described by Hall and Moraru [9], which was adapted from AACC method 56–30. Five g of samples were centrifuged at 2000× *g* for 10 min at 20 °C. After decanting the supernatant, the sample was weighed and water holding capacity was determined as follows:

(1) $$WHC=\frac{Initial\;weight\;of\;sample-Supernatant\;weight}{Initial\;weight\;of\;sample}\times 100\%$$

Water holding capacity measurements reported in this study represent the water separating from the gel sample after centrifugation, not the spontaneous syneresis that may have occurred prior to analysis.

### 4.11. Spontaneous Syneresis

Spontaneous syneresis in self-standing gel samples was determined by the following after 1 h of processing and within 10 min of each other by weighing the expelled liquid from the gel sample:

(2) $$Syneresis=\frac{Exceeded\;water\;weight}{Initial\;weight\;of\;sample}\times 100\%$$

### 4.12. Scanning Electron Microscopy (SEM)

The microstructures of select PPC gels that had different rheological and textural behavior were assessed by scanning electron microscopy (SEM). Gels were cut into 1 mm thick slices, frozen in liquid nitrogen (−196 °C), and freeze-dried for 20 h (Labconco, Kansas City, MO, USA). Dried specimens were mounted on SEM aluminum stubs, painted with silver paint to create a conductive path to the surface of the SEM stub, sputter-coated with gold-palladium using a high-vacuum coater (Denton Desk V, Pfeiffer, Germany), and evaluated with a scanning electron microscope (ZEISS Gemini 500, ZEISS, Oberkochen, Germany) operating at 1 kV. SmartSEM software (Carl Zeiss Microscopy, LLC, Hamburg, Germany) was used to acquire images. Images with widths of 30, 200, and 500 µm were taken for comparison.

### 4.13. Macroscopic Visual Examination of Gel Cross-Section

To assess the macroscopic structure of HPP- and thermally processed samples, photographs of gel cross-sections (diameter of 24 mm) were taken.

### 4.14. Statistical Analysis

Each sample type was prepared and processed independently in biological triplicate. Reported average and standard error values were calculated based on these independent replicates. Particle size, zeta potential, free Ca^2+^, pH, rheological, and texture profile analyses were each conducted in at least technical triplicate, and syneresis and water holding capacity analyses were conducted in technical duplicates. Data was analyzed for statistical significance using JMP Pro 16 (SAS Institute Inc., Cary, NC, USA) by fitting data to a linear model that modeled each analysis by protein type with main effects of process type, acidification level, and calcium addition, as well as all two-way and three-way interactions, and random effects for biological replicates and for technical replicates nested within biological replicates. Significant differences were determined at α = 0.05 and pairwise comparisons were made using a post hoc Tukey–Kramer HSD multiple comparison test.

## Figures and Tables

**Table 1:** Water holding capacity (%) of unprocessed, HPP-, and thermally processed pea protein concentrate (PPC), lentil protein concentrate (LPC), and faba bean protein concentrate (FPC) samples at different acidification and calcium addition levels. Values represent averages of independent biological triplicates ± 1 standard error, each replicate being an average of technical duplicates. Different lowercase letters in a row for a protein type indicate significant differences (*p* < 0.05) between unprocessed, HPP-, and thermally processed samples of a given type. Different uppercase letters indicate significant differences between samples with varying acidification and calcium levels.

Acidification	Calcium (mg Ca/g Protein)	Water Holding Capacity (%)								
		PPC			LPC			FPC		
		Unprocessed	HPP	Thermal	Unprocessed	HPP	Thermal	Unprocessed	HPP	Thermal
*No Acid*	*0*	41.6 ± 1.7 b, E	94.2 ± 0.7 a, A	100 ± 0 a, A	40.3 ± 0.7 b, F	94.3 ± 0.8 a, A	100 ± 0 a, A	35.6 ± 0.1 c, D	92.8 ± 1.6 b, AB	100 ± 0 a, A
	*10*	65.5 ± 1.2 c, CD	91.3 ± 2.3 b, A	100 ± 0 a, A	56.9 ± 3.4 c, E	90.2 ± 1.7 b, A	100 ± 0 a, A	50.8 ± 1.3 c, C	87.4 ± 1.1 b, C	100 ± 0 a, A
	*20*	68.1 ± 2.9 b, BCD	94.2 ± 2.7 a, A	99.8 ± 0.2 a, A	71.6 ± 2.8 b, ABC	91.9 ± 1.3 a, A	100 ± 0 a, A	55 ± 2.9 c, BC	92 ± 1.5 b, ABC	100 ± 0 a, A
	*30*	74.3 ± 3.1 b, AB	93.2 ± 2 a, A	99.9 ± 0.1 a, A	62 ± 10.4 b, DE	95.9 ± 1 a, A	100 ± 0 a, A	67.1 ± 0.8 c, A	90.2 ± 2.6 b, ABC	100 ± 0 a, A
*Low Acid*	*0*	64.2 ± 5.1 c, D	91.6 ± 2 b, A	100 ± 0 a, A	56 ± 5.2 c, E	88.5 ± 0.9 b, A	100 ± 0 a, A	55.5 ± 0.4 c, BC	88 ± 1.1 b, BC	100 ± 0 a, A
	*10*	65.3 ± 4.3 b, D	95.4 ± 2.2 a, A	100 ± 0 a, A	65.5 ± 1.4 c, CD	89.9 ± 0.9 b, A	100 ± 0 a, A	53.1 ± 0.8 c, BC	89.4 ± 1.3 b, ABC	100 ± 0 a, A
	*20*	68.3 ± 3 b, BCD	94 ± 2.8 a, A	99.9 ± 0.1 a, A	72.5 ± 1.5 b, ABC	92.3 ± 0.7 a, A	100 ± 0 a, A	54.8 ± 1.6 c, BC	93.3 ± 2.7 b, A	100 ± 0 a, A
	*30*	77 ± 3.8 b, A	95.2 ± 2.7 a, A	100 ± 0 a, A	78.9 ± 1.4 b, A	93.1 ± 0.6 a, A	100 ± 0 a, A	64.3 ± 1.4 c, A	93.8 ± 1.4 b, A	100 ± 0 a, A
*High Acid*	*0*	67.1 ± 2.1 b, BCD	96.7 ± 2.2 a, A	100 ± 0 a, A	66.6 ± 1.8 b, CD	93 ± 0.6 a, A	100 ± 0 a, A	55.4 ± 0.8 c, BC	92.1 ± 2 b, ABC	100 ± 0 a, A
	*10*	69.7 ± 1.5 b, ABCD	95.1 ± 2.8 a, A	100 ± 0 a, A	69 ± 0.7 b, BCD	93.2 ± 1 a, A	100 ± 0 a, A	55.8 ± 0.7 c, BC	92.5 ± 2 b, ABC	100 ± 0 a, A
	*20*	73.7 ± 3.4 b, ABC	96.4 ± 2.2 a, A	100 ± 0 a, A	72.6 ± 1.1 b, ABC	93.4 ± 0.9 a, A	100 ± 0 a, A	58.2 ± 0.8 c, BC	90.9 ± 1.8 b, ABC	100 ± 0 a, A
	*30*	74.2 ± 3.3 b, AB	95.6 ± 2.4 a, A	100 ± 0 a, A	77 ± 1.6 b, AB	93.3 ± 0.3 a, A	100 ± 0 a, A	63.6 ± 2.6 c, A	90.3 ± 1.3 b, ABC	100 ± 0 a, A

**Table 2:** General trends in gel properties (gel strength, hardness, cohesiveness, and syneresis) for PPC, LPC, and FPC gels processed by HPP or thermal treatment. Gels of each process type are compared across three treatments [Acidification (High Acid), Ca Addition (30 mg Ca/g protein), and Acid + Ca (High Acid, 30 mg Ca/g protein)] relative to their respective control (No Acid, 0 mg Ca/g protein). Symbols indicate direction of effect: increase (↑), decrease (↓), no changes (-).

Sample	Process	Additional Treatment	Gel Strength (G′)	Gel Hardness	Gel Cohesiveness	Syneresis Post-Process
Pea (PPC)	HPP	Acidification	↑↑	↑↑	-	↑↑
		Ca addition	↑	↑	↓	-
		Acid + Ca	↑↑	↑↑	-	↑↑
	Thermal	Acidification	↑↑	-	↓	None observed
		Ca addition	-	↓	↓	None observed
		Acid + Ca	↑	-	↓	None observed
Lentil (LPC)	HPP	Acidification	↑↑	↑↑	↓	↑↑
		Ca addition	↑	↑	↓	↑
		Acid + Ca	↑	↑	↓	↑
	Thermal	Acidification	↑↑	↑	↓	None observed
		Ca addition	-	↓	↓	None observed
		Acid + Ca	↑	-	↓	None observed
Faba (FPC)	HPP	Acidification	↑↑	↑↑	-	↑↑
		Ca addition	↑	↑	↓	-
		Acid + Ca	↑↑	↑↑	-	↑↑
	Thermal	Acidification	↑	-	↓	None observed
		Ca addition	-	↓↓	↓	None observed
		Acid + Ca	↑	↑	↓	None observed

**Table 3:** Composition of pea protein concentrate (PPC), lentil protein concentrate (LPC), and faba bean protein concentrate (FPC) as determined by Dairy One (Ithaca, NY, USA).

	PPC	LPC	FPC
Macronutrients (g/100 g)			
Protein	52.8	49.0	60.1
Simple Sugars	11.8	8.9	7
Starch	5.7	18.9	6.3
Moisture	8.2	7.6	7.8
Crude Fat	2.66	1.7	1.77
Ash	5.64	4.94	6.66
Minerals (mg/100 g)			
Sodium	10	7	17
Potassium	1690	1480	1970
Calcium	100	40	90
Sulfur	390	340	370
Chloride	120	110	100
Magnesium	230	130	230
Phosphorus	750	670	930
Iron	5.0	6.9	5.9
Zinc	5.9	6.5	10.1
Copper	1.6	1.7	2.1
Manganese	2.1	2.0	2.0

**Table 4:** Composition of pea protein concentrate (PPC), lentil protein concentrate (LPC), and faba bean concentrate (FPC) samples calculated from the composition of PPC, LPC, and FPC powders when prepared at a protein concentration of 15 g protein/100 g. Values represent g per 100 g sample.

Sample	Protein (g/100 g)	Starch (g/100 g)	Crude Fat (g/100 g)	Ash (g/100 g)	Total Solids (g/100 g)
PPC	15.00	1.62	0.76	1.60	26.08
LPC	15.00	5.79	0.52	1.51	28.32
FPC	15.00	1.57	0.44	1.66	23.01

## Data Availability

The data can be made available upon direct request to the authors.

## Supplementary Materials

The following supporting information can be downloaded at https://www.mdpi.com/article/10.3390/gels11120971/s1: Table S1. ζ-potential of unprocessed PPC, LPC, and FPC suspensions at different acidification and calcium addition levels. Table S2. Effective diameter of particles in unprocessed PPC, LPC, and FPC suspensions, at different acidification and calcium addition levels. Table S3. Free Ca^2+^ concentration of unprocessed PPC suspensions at different acidification and calcium addition levels. Figure S1. pH of unprocessed pea protein concentrate (PPC), lentil protein concentrate (LPC), and faba bean protein concentrate (FPC) suspensions at different acidification and calcium addition levels. Figure S2. Example particle size distribution (intensity vs. diameter) of unprocessed PPC suspensions at different acidification and calcium addition levels. Figure S3. Examples of strain sweeps of (a) PPC, (b) LPC, and (c) FPC of Thermally processed No Acid-0 mg Ca/g protein gels, showing G′, G″, and tanδ vs. strain. Figure S4. Storage modulus, G′ vs. frequency for unprocessed, HPP-, and thermally processed PPC (a–c), LPC (d–f), and FPC (g–i) samples at different acidification and calcium addition levels. Figure S5. Scanning electron microscopy (SEM) images of HPP- and thermally processed pea protein concentrate gels at No Acid-0 mg Ca/g protein, No Acid-30 mg Ca/g protein, and High Acid-0 mg Ca/g protein. Figure S6. Photographs of faba bean protein concentrate gels that were thermally processed (95 °C, 15 min) and HPP (600 MPa, 5 °C, 4 min).

## Abbreviations

The following abbreviations are used in this manuscript:

PPC	Pea protein concentrate
LPC	Lentil protein concentrate
FPC	Faba bean protein concentrate
HPP	High-pressure processing
