# Supplementary material for: Acidification and Calcium Addition Effects on High-Pressure and Thermally Induced Pulse Protein Gels
Source: Gels. 2025 Dec 2;11(12):971. doi: 10.3390/gels11120971 (PMC12732794; doi:10.3390/gels11120971)
Supplement: Supplementary file 1 [file gels-11-00971-s001.zip › gels-3973152-supplementary.pdf]

Acidification and Calcium Addition Effects on High Pressure and Thermally Induced Pulse Protein Gels

April Huang and Carmen I. Moraru, Cornell University

Supplemental Material

Table S1. ζ-potential of unprocessed PPC, LPC, and FPC suspensions at different acidification and calcium addition levels. Values are the averages and standard error of independent biological replicates, which are each an average of technical triplicates. PPC and LPC samples each have biological triplicates while FPC samples have biological duplicates.

Acidification level	Calcium addition (mg Ca/g protein)	Protein type					
		PPC		LPC		FPC	
		ζ-potential mean (mV)	Std Err (mV)	ζ-potential mean (mV)	Std Err (mV)	ζ-potential mean (mV)	Std Err (mV)
No Acid	0	-26.5	0.7	-27.4	1.7	-26.2	0.5
	10	-20.0	1.4	-18.5	1.8	-20.1	0.6
	20	-17.1	1.0	-14.2	1.7	-11.5	2.9
	30	-16.5	2.5	-13.3	1.3	-14.0	1.9
Low Acid	0	-23.4	0.9	-18.4	0.8	-20.1	0.8
	10	-17.1	1.2	-12.1	0.8	-16.2	0.3
	20	-14.1	0.6	-10.2	0.5	-10.6	2.5
	30	-13.4	1.0	-7.1	0.4	-9.3	1.2
High Acid	0	-8.0	0.6	-3.6	1.3	-3.2	3.1
	10	-6.2	0.8	1.0	1.9	-4.4	0.3
	20	-3.5	0.7	3.7	2.9	-3.9	1.7
	30	-1.5	1.1	3.9	1.2	-1.9	1.1

Table S2. Effective diameter of particles in unprocessed PPC, LPC, and FPC suspensions, at different acidification and calcium addition levels. Values are the averages and standard error of independent biological replicates, which are each an average of technical triplicates. PPC and LPC samples each have biological triplicates while FPC samples have biological duplicates.

Acidification level	Calcium addition (mg Ca/g protein)	Protein type					
		PPC		LPC		FPC	
		Effective diameter, mean (nm)	Std Err (nm)	Effective diameter, mean (nm)	Std Err (nm)	Effective diameter, mean (nm)	Std Err (nm)
No Acid	0	739.1	43.2	505.1	85.1	603.3	104.8
	10	1119.0	240.9	922.2	183.2	849.0	93.3
	20	1589.5	265.6	*	*	1150.8	158.1
	30	1682.1	311.6	*	*	*	*
Low Acid	0	1042.7	128.0	867.8	185.0	1134.6	254.4
	10	1824.6	267.7	1125.3	398.8	885.1	296.5
	20	1761.8	140.8	*	*	815.2	62.4
	30	2896.5	265.0	*	*	*	*
High Acid	0	2491.3	707.9	1794.1	1022.4	1182.1	245.0
	10	2672.1	278.0	*	*	1055.8	365.7
	20	2535.9	382.0	*	*	*	*
	30	2576.7	607.7	*	*	*	*

**Data not available due to the formation of large, visible aggregates (see picture below as example)*

Images showing dilute samples of FPC with No Acid-0 mg Ca/g protein (left) and FPC with Low Acid-30 mg Ca/g protein (right) showing visible aggregation:

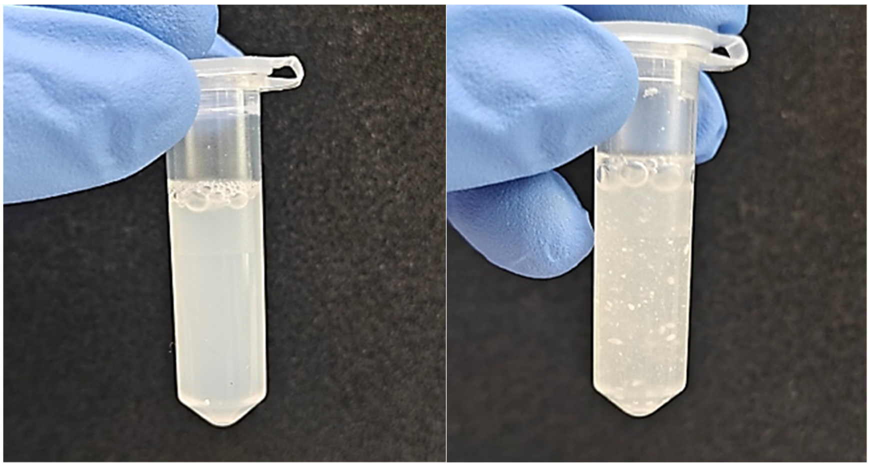

Table S3. Free Ca^{2+} concentration of unprocessed PPC suspensions at different acidification and calcium addition levels. Values are the averages and standard error of independent biological replicates, which are each an average of technical triplicates. PPC samples have biological triplicates while LPC and FPC samples have biological duplicates.

Acidification level	Calcium addition (mg Ca/g protein)	Protein type					
		PPC		LPC		FPC	
		Free [Ca^{2+}] (mM) Mean	Std Err (mM)	Free [Ca^{2+}] (mM) Mean	Std Err (mM)	Free [Ca^{2+}] (mM) Mean	Std Err (mM)
No Acid	0	0.2	0.0	0.0	0.0	0.0	0.0
	10	2.6	0.1	2.7	0.2	1.4	0.2
	20	10.9	0.2	11.8	0.6	7.1	1.0
	30	25.6	0.3	32.2	3.2	21.7	2.3
Low Acid	0	0.3	0.0	0.1	0.0	0.2	0.0
	10	4.2	0.1	5.4	0.8	3.4	0.5
	20	13.7	0.2	19.8	2.3	11.1	1.4
	30	27.3	0.5	43.1	3.6	25.3	2.4
High Acid	0	0.5	0.0	0.3	0.0	0.4	0.0
	10	5.7	0.1	8.4	0.7	4.9	0.5
	20	16.7	0.2	27.6	2.9	13.9	1.3
	30	31.7	0.6	54.4	2.9	31.2	2.7

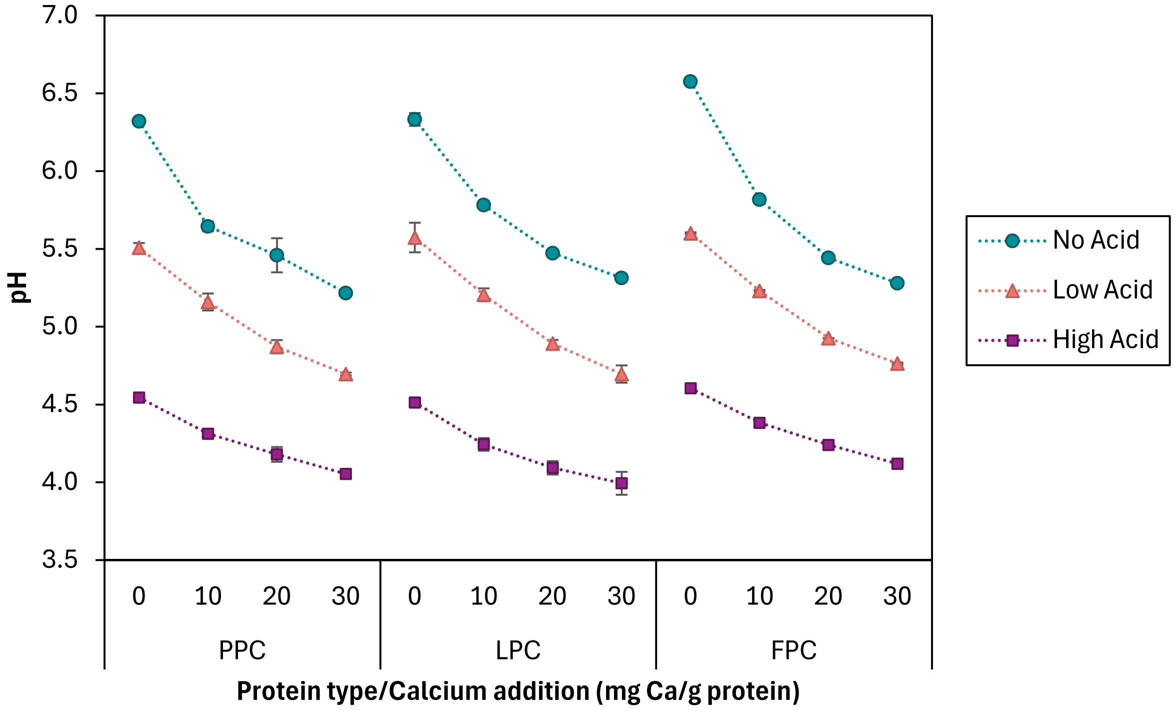

Figure S1. pH of unprocessed PPC, LPC, and FPC suspensions at different acidification and calcium addition levels. Values represent averages of independent biological triplicates, which are each an average of technical triplicates. Error bars represent ± 1 standard error.

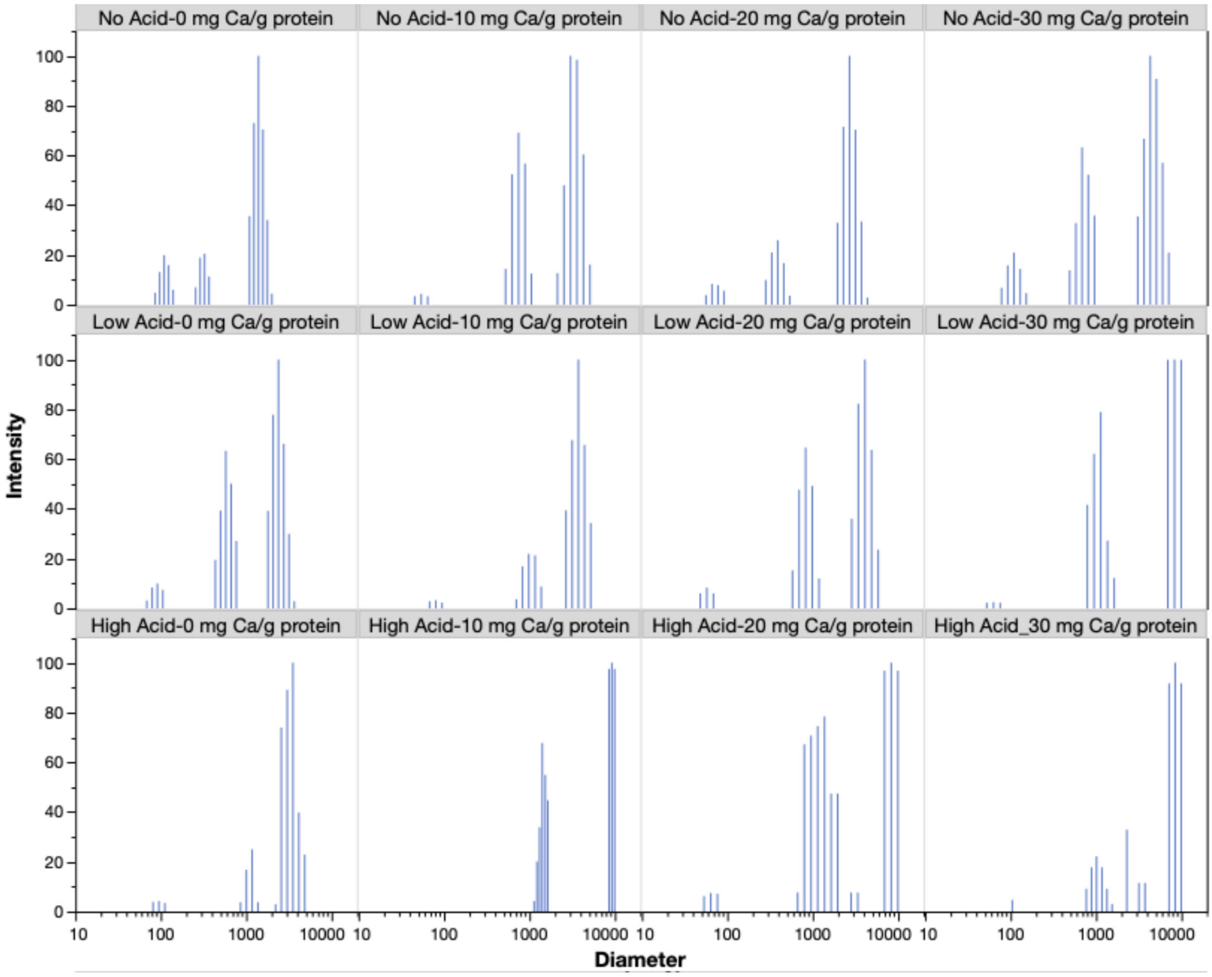

Figure S2. Example particle size distribution (intensity vs. diameter) of unprocessed PPC suspensions at different acidification and calcium addition levels.

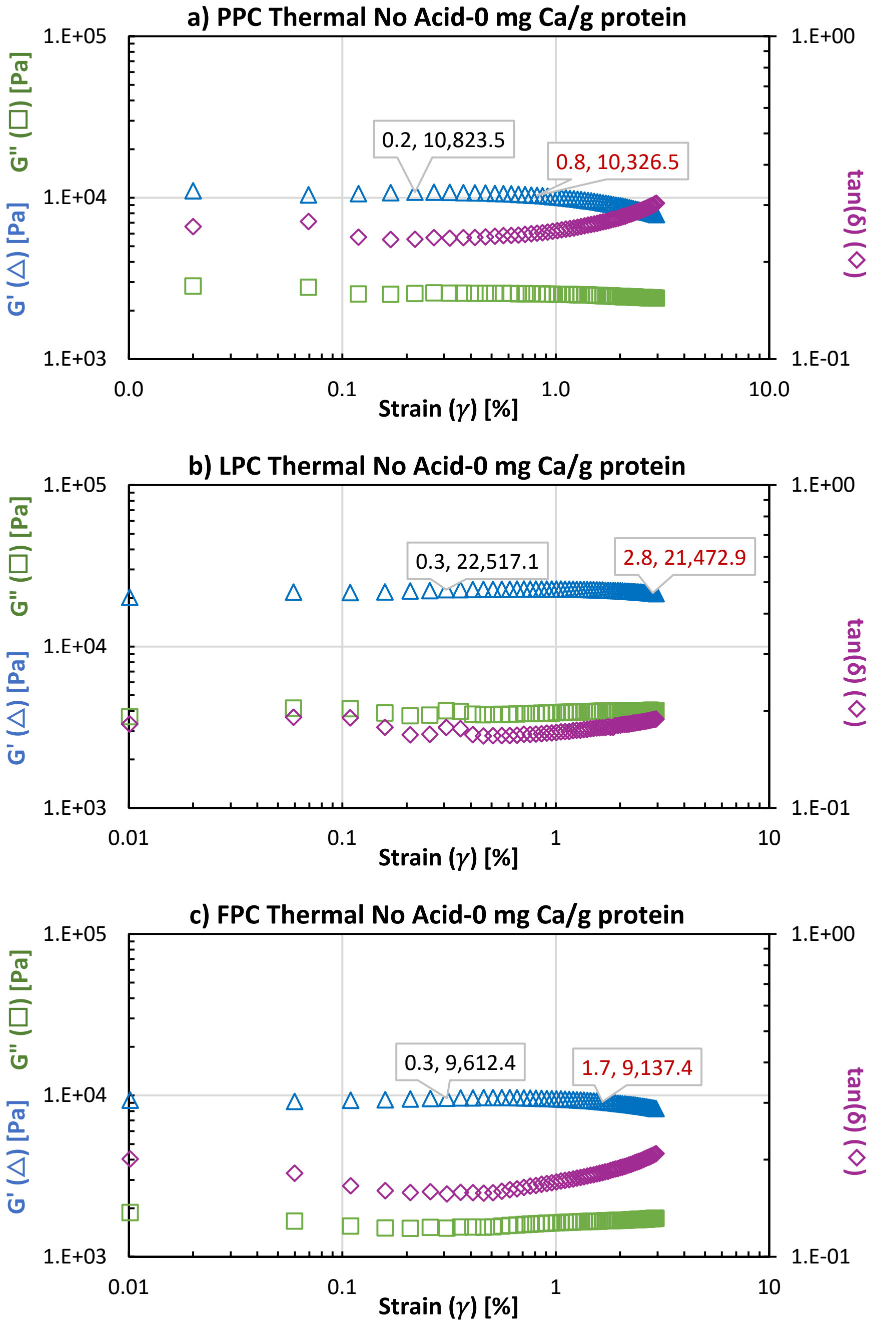

Figure S3. Examples of strain sweeps of a) PPC, b) LPC, and c) FPC of Thermally processed No Acid-0 mg Ca/g protein gels, showing G′, G″, and tanδ vs strain. The critical strain (data point labeled in red font) is the end of the linear viscoelastic region where the G′ begins drops by ~5% compared to the G′ in the plateau region (data point labeled in black font). For frequency sweeps, a strain value below the critical strain value that gives adequate signal (torque above minimum value) is chosen. In the case of self-standing gel samples, a strain of 0.5% was used. In free-flowing samples, a strain of 0.5-1% was used.

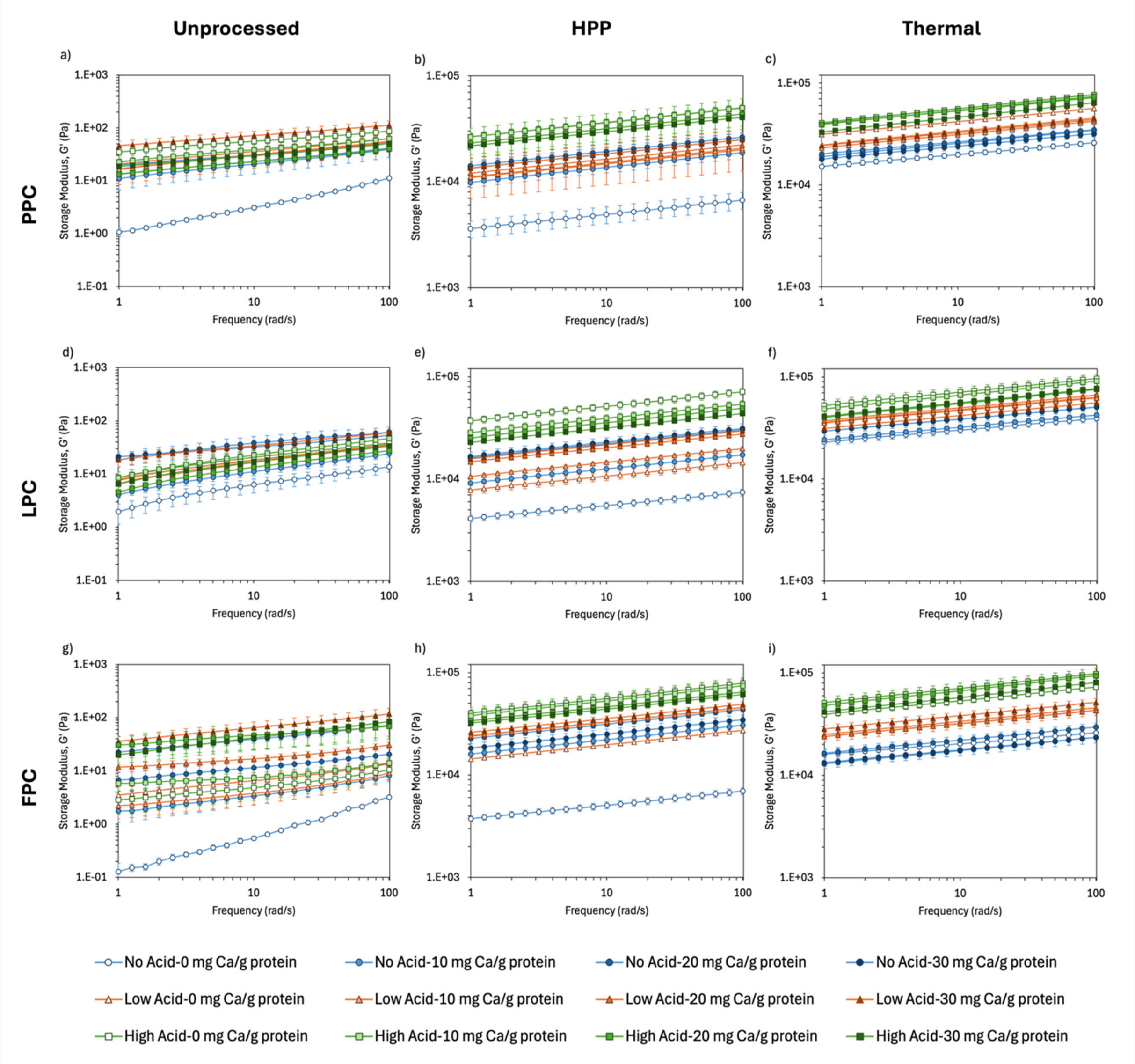

Figure S4. Storage modulus, G′ vs. frequency for unprocessed, HPP, and thermally processed PPC (a-c), LPC (d-f), and FPC (g-i) samples at different acidification and calcium addition levels. Values represent averages of independent biological triplicates, which are each an average of technical triplicates. Error bars represent ± 1 standard error. Since G′ values for unprocessed samples were several orders of magnitude lower than those for processed samples, the y-axis scale for these samples differs from that used for processed samples.

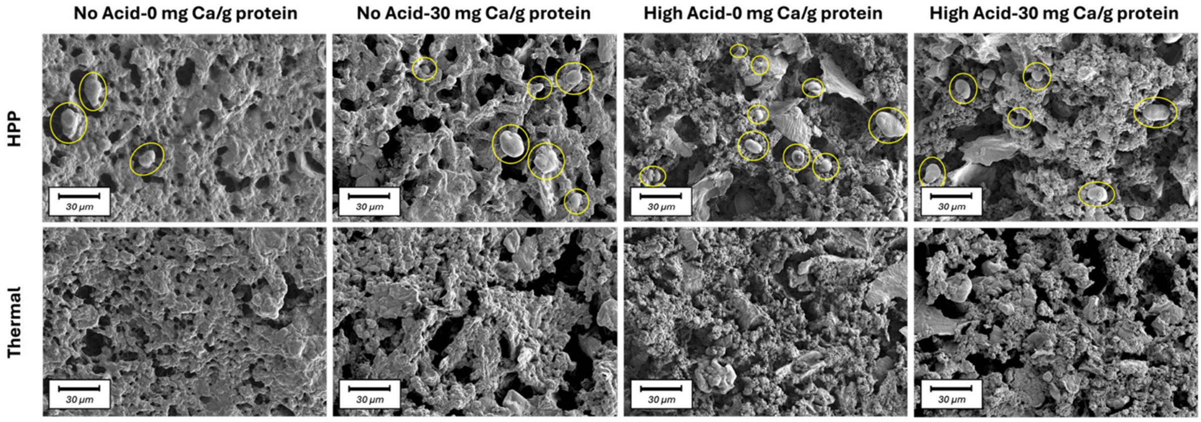

Figure S5. Scanning electron microscopy (SEM) images of HPP and thermally processed PPC gels at No Acid-0 mg Ca/g protein, No Acid-30 mg Ca/g protein, and High Acid-0 mg Ca/g protein. Scale bars represent 30 μm. Yellow circles indicate starch granules.

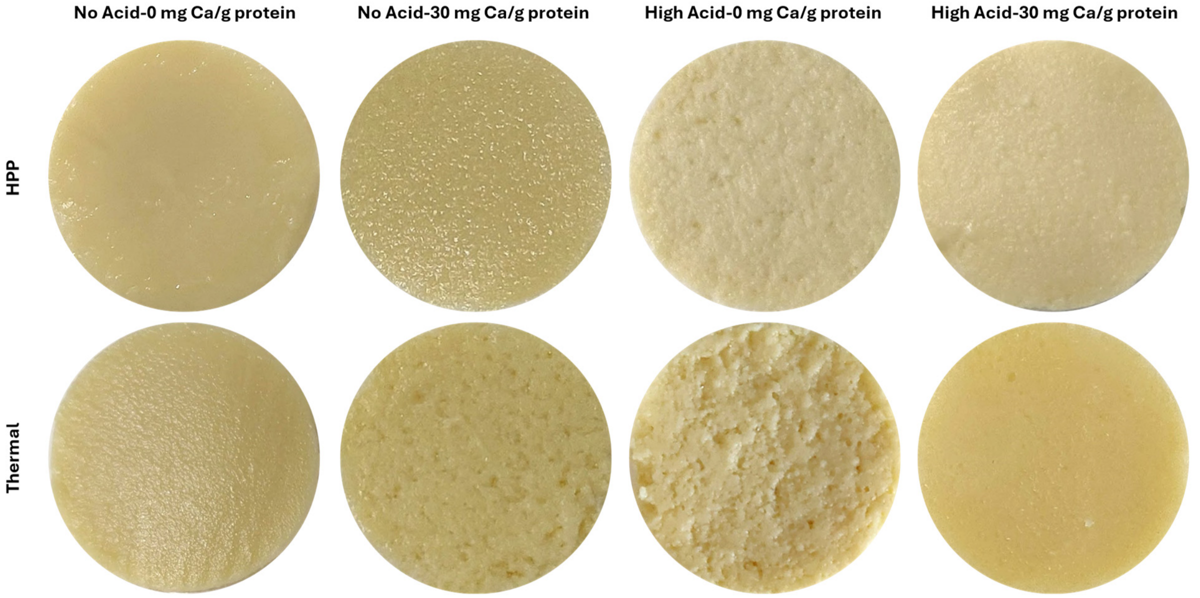

Figure S6. Photographs of HPP and thermally processed FPC gels at No Acid-0 mg Ca/g protein, No Acid-30 mg Ca/g protein, and High Acid-0 mg Ca/g protein. Each sample was 25 mm in diameter.
